# LEOPARD syndrome with accelerated idioventricular rhythm and systolic anterior motion of the posterior mitral leaflet: a case report

**DOI:** 10.1093/ehjcr/ytae309

**Published:** 2024-06-29

**Authors:** Naotoshi Wada, Shoji Keisuke, Tetsuya Nomura, Natsuya Keira, Tetsuya Tatsumi

**Affiliations:** Department of Cardiovascular Medicine, Kyoto Chubu Medical Center, 25, Yagi-Ueno, Yagi-cho, Nantan City, Kyoto 629-0197, Japan; Department of Cardiovascular Medicine, Kyoto Chubu Medical Center, 25, Yagi-Ueno, Yagi-cho, Nantan City, Kyoto 629-0197, Japan; Department of Cardiovascular Medicine, Kyoto Chubu Medical Center, 25, Yagi-Ueno, Yagi-cho, Nantan City, Kyoto 629-0197, Japan; Department of Cardiovascular Medicine, Kyoto Chubu Medical Center, 25, Yagi-Ueno, Yagi-cho, Nantan City, Kyoto 629-0197, Japan; Department of Cardiovascular Medicine, Kyoto Chubu Medical Center, 25, Yagi-Ueno, Yagi-cho, Nantan City, Kyoto 629-0197, Japan

**Keywords:** Case report, LEOPARD syndrome, Accelerated idioventricular rhythm, Systolic anterior motion of the posterior mitral leaflet, PTPN11 gene variant

## Abstract

**Background:**

PTPN11 is ubiquitously expressed and has a variety of phenotypes even in a single heart. We examined LEOPARD syndrome (LS) in a patient with *PTPN11* variants through pathological, electrophysiological, and anatomical studies.

**Case summary:**

A 49-year-old man with no previous medical history was brought to our emergency department because of syncope. An electrocardiogram (ECG) revealed alternating bundle branch block, and echocardiography revealed hypertrophic cardiomyopathy-like morphology with systolic anterior motion of the posterior mitral valve. Atrioventricular block, left ventricular outflow tract (LVOT) obstruction, and ventricular tachycardia were considered the differential diagnoses; however, the treatment plan was difficult to determine. An electrophysiological study revealed the cause of the ECG abnormality to be accelerated idioventricular rhythm, and the programmed ventricular stimulation was negative. Genetic testing revealed LS with *PTPN11* variant, which was speculated to be the cause of these various unique cardiac features. The cause of syncope was considered to be exacerbation of LVOT obstruction due to dehydration, and the patient was treated with oral beta-blockers. Implantable loop recorder observation for 1 year revealed no arrhythmia causing syncope, and an implantable cardioverter-defibrillator and pacemaker were deemed unnecessary for primary prevention of syncope. During 2.5 years of follow-up, the LVOT peak velocity fluctuated between 2.5 and 3.5 m/s, but the patient remained stable with no recurrent syncope.

**Conclusion:**

We confirmed that LS is distinct from other cardiomyopathies using characterization, physiological, electrophysiological, and pathological examinations. Evidence supporting a specific treatment strategy for LS is limited, and understanding the pathogenesis may help establish effective treatment strategies.

**Learning points**

- LEOPARD syndrome can present with syncope in adulthood.
- Various cardiac phenotypes may complicate the diagnosis and determination of treatment strategies.
- Cases of LEOPARD syndrome can present with accelerated idioventricular rhythm and left ventricular outflow tract obstruction with systolic anterior motion of the posterior mitral valve.
- Physiological, electrophysiological, and pathological examinations can help distinguish LEOPARD syndrome from other cardiomyopathies.

## Introduction

LEOPARD syndrome (LS), a type of RAS/MAPK pathway disorder (RASopathies), is a malformation syndrome named by Gorlin in 1969 and an acronym for lentigines, electrocardiogram (ECG) conduction abnormalities, ocular hypertelorism, pulmonic stenosis, abnormal genitalia, retardation of growth, and deafness.^1^ About 85% of patients with LS show variants in exons 7, 12, and 13 of the *PTPN11* gene.^2^ PTPN11 encodes a Src homology region 2 (SH2) domain–containing tyrosine phosphatase 2 or SHP2 protein. SHP2 is a molecule involved in cellular responses to growth factors, hormones, cytokines, and cell adhesion molecules. Shp2 is a ubiquitously expressed non-receptor protein tyrosine phosphatase (PTP) consisting of two N-terminal SH2 domains, a catalytic (PTP) domain, and a C-terminus with tyrosyl phosphorylation sites and a proline-rich stretch. Shp2 binds directly to growth factor receptors, such as platelet-derived growth factor (PDGF) receptors, and several scaffolding adaptors including IRS, FGF receptor substrate (FRS), and Gab proteins via its SH2 domain, and is considered necessary for full activation of most Ras/Erk cascades. Functional analysis indicates that Noonan syndrome (NS) is caused by gain-of-function *PTPN11* variants, whereas LS is caused by autosomal dominant loss-of-function variant.^3^ The *PTPN11* variant (p.Gly464Ala) does not increase *PTPN11* activity and is described as a specific causative variant in LS due to the increased bulk of the alanyl side chain caused by Gly464Ala, which blocks the access of the substrate phosphorylation group to the active site. Common cardiac abnormalities observed in RASopathies include hypertrophic cardiomyopathy (HCM), valve abnormalities, especially pulmonary valve stenosis, and dysplasia, with a particularly high reported incidence of atrial and ventricular septal defects.^4^ In electrophysiological phenotypes, non-reentrant, multifocal, and ectopic atrial tachycardias have been reported in patients with RASopathies.^5^ However, in the absence of large-scale population studies, the pathogenesis of heart disease remains unclear. The diagnosis is based on characteristic physical findings, family history, and genetic testing with echocardiography, electrocardiography, and hearing loss sometimes serving as auxiliary diagnostic tests. However, prenatal genetic testing is not covered by medical insurance in Japan, and the diagnosis is not reached in some cases. Physical findings characteristic of LS in childhood are often not confirmed as abnormal findings in adults, and the diagnosis is often difficult. In this study, we experienced a case of solitary LS that was first diagnosed in adulthood following syncope. In this case, HCM was accompanied by accelerated idioventricular rhythm (AIVR) and left ventricular outflow tract (LVOT) obstruction with systolic anterior motion (SAM) of the posterior mitral valve (PMV), which have not been reported in the past literature. The various cardiac phenotypes sometimes complicate diagnosis and treatment strategies and require careful observation.

## Summary figure

**Table:** 

Time	Events
Day 1	A 49-year-old man was transported to the emergency room with loss of consciousness and head trauma
10 days later	Implantable loop recorder (ILR) placed in the body
24 days later	Coronary angiography, electrophysiological study, and endomyocardial biopsy were performed on admission
3 months later	Negative head-up tilt test ruled out neurally mediated syncope
4 months later	Based on the results of the genetic search, he was diagnosed with LEOPARD syndrome.
5 months later	A beta-blocker was prescribed
12 months later	Since there was no recurrence of syncope, the ILR was removed.

## Case presentation

**Figure 1:**
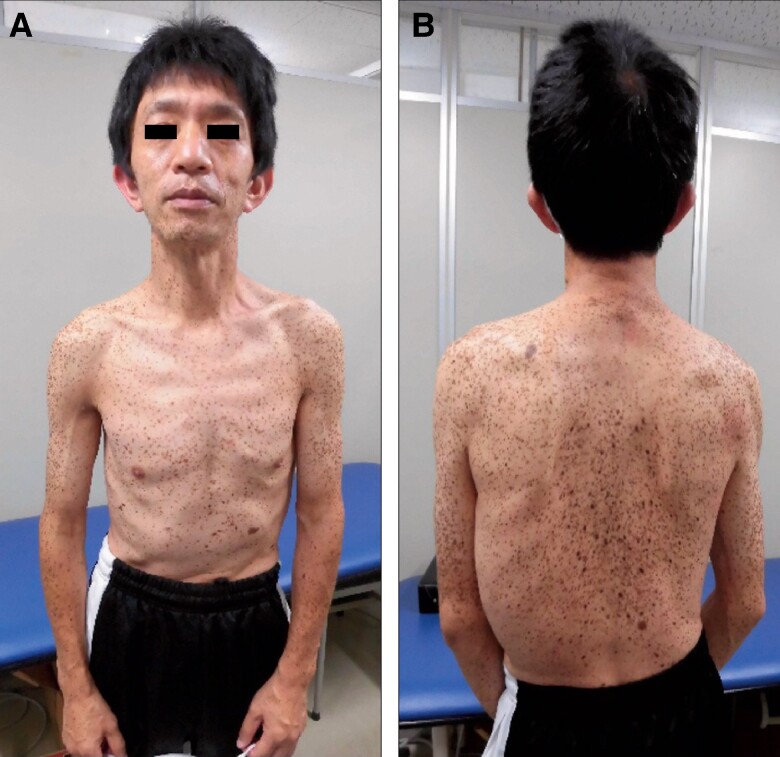
Physical examination shows dysmorphic features: (*A)* Multiple lentigines are scattered from the front of the trunk to the neck, low-set ears, flat nasal bridge, and pectus excavatum. *(B)* Multiple lentigines are scattered over the back of the trunk.

**Figure 2:**
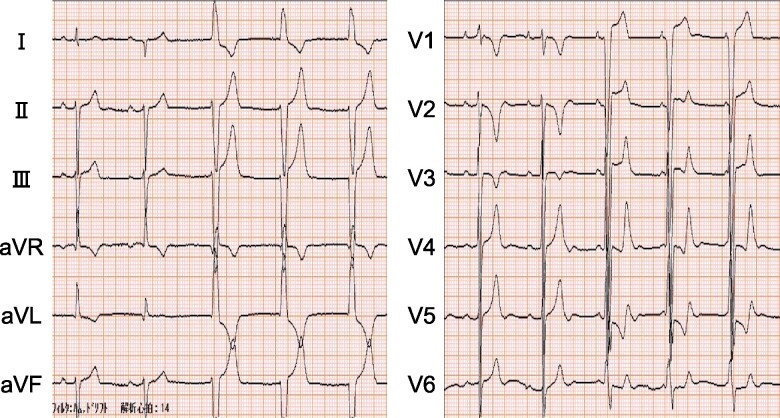
Electrocardiogram shows an incomplete right bundle branch block for the first two beats followed by three beats of accelerated idioventricular rhythm.

**Figure 3:**
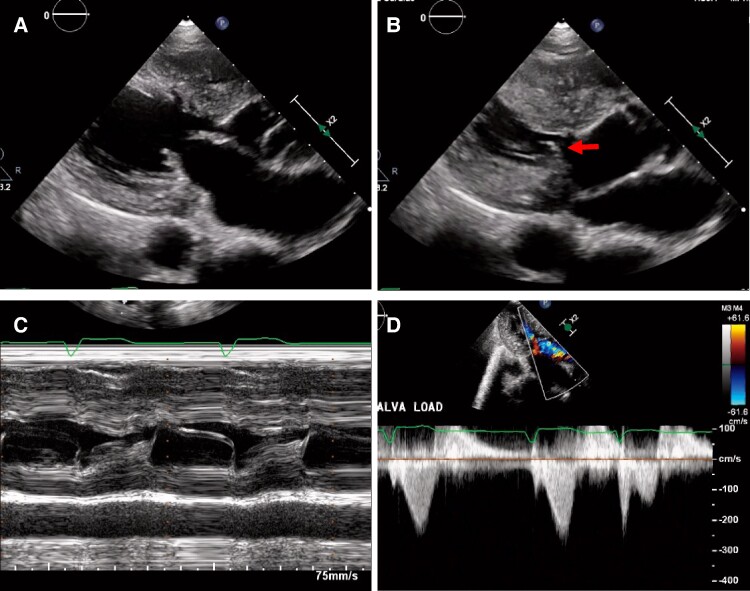
Echocardiography: (*A)* Mid-diastolic image shows hypertrophy of the left ventricular septum (16 mm) and a left ventricular non-compaction–like structure. *(B)* Systolic image shows the posterior mitral valve moving to contact the left ventricular septum (arrows). *(C)* M-mode image shows systolic anterior motion of the posterior mitral valve. *(D)* Doppler image shows left ventricular outflow tract obstruction of 31 mmHg by the Valsalva manoeuver.

A 49-year-old man lost consciousness following a head injury while drying clothes in industrial clothes drying room and was brought to the hospital emergency room by ambulance. He had no particular medical history and was not prescribed any medications at the time of arrival. Upon arrival at the hospital, he was conscious and did not exhibit any concussion symptoms such as headache, neck pain, nausea or vomiting, dizziness, confusion, difficulty concentrating, difficulty remembering, or drowsiness. His blood pressure was 90/67 mmHg, heart rate was 69 b.p.m., and oxygen saturation was 99% on room air. Physical examination revealed ocular hypertelorism, low-set ears, a flat nasal bridge, pectus excavatum, and multiple lentigines (*Figure 1*). An ECG revealed a heart rate of 71 b.p.m. and incomplete right bundle branch block for the first two beats, followed by accelerated idioventricular rhythm (*Figure 2*). Chest radiography showed no abnormalities. Echocardiography revealed asymmetric septal hypertrophy and a left ventricular non-compaction–like structure. The posterior mitral leaflet was elongated to 17 mm, and the Valsalva manoeuver resulted in an accelerated blood flow of 2.8 m/s at the LVOT associated with the SAM of the PMV (*Figure 3*). There were no abnormalities of the papillary muscles. At this point, the present illness was suspected to be non-traumatic transient loss of consciousness, and atrioventricular block, ventricular tachycardia (VT) associated with HCM, LVOT obstruction, and neurally mediated syncope were considered as differential diseases for syncope. Since there was a risk of recurrence, we recommended hospitalization at the emergency room. However, he refused to be hospitalized due to his work schedule; therefore, we decided to follow the patient in an outpatient department. Since ventricular arrhythmia associated with HCM could not be ruled out, we applied continuous heart rhythm monitoring with an ILR in an outpatient setting 10 days later. Blood tests and cardiac magnetic resonance imaging showed no findings suggesting secondary cardiomyopathy such as HCM, cardiac amyloidosis, or ischaemic cardiomyopathy. A negative head-up tilt test with a beta stimulant in the outpatient setting excluded neurally mediated syncope. No hearing loss was noted, although audiometry was performed.

**Figure 4:**
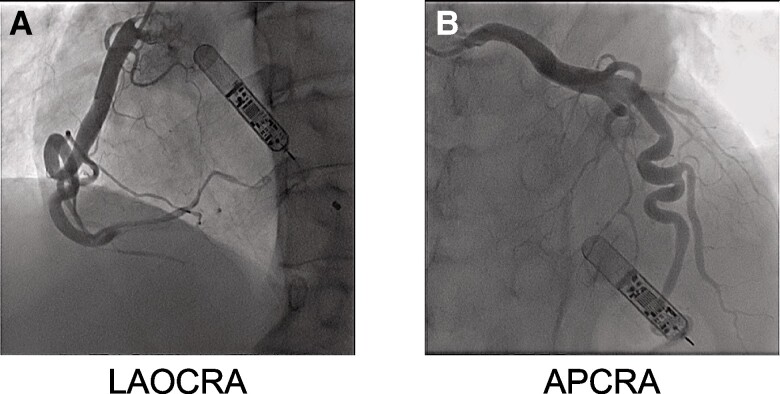
Coronary angiography: *(A)* Ectasia and tortuosity of the right coronary artery. *(B)* Ectasia and tortuosity of the left coronary artery.

**Figure 5:**
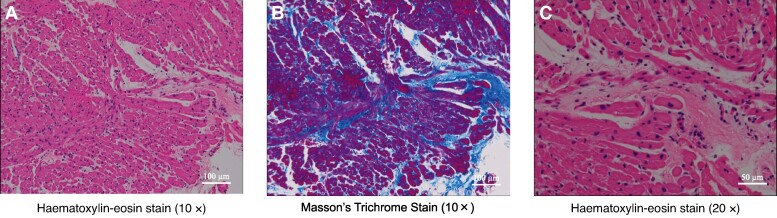
Histological findings: *(A)* Histological section of the right ventricular septum. *(B)* Masson trichrome staining shows interstitial fibrosis. *(C)* Haematoxylin-eosin stain prominently shows a small myocardium.

After 24 days and 1 night of hospitalization after the initial visit, coronary angiography showed ectasia of the left and right coronary arteries; however, there were no significant stenotic lesions (*Figure 4*). Subsequently, electrophysiological study showed AIVR of ∼75 b.p.m., although the programmed ventricular stimulation was negative. The histological findings of the myocardial biopsy showed interstitial fibrosis, no evidence of myocyte hypertrophy or myocyte disarray seen in the HCM, and conspicuous small myocytes (*Figure 5*). With the patient’s consent, Orphan Net Japan Kazusa screened for variants in *PTPN11*, *SOS1*, *RAF1*, *RITI*, *KRAS*, *NRAS*, *SHOC2*, *CBL*, *BRAF*, *HRAS*, *MAP2K1*, and *MAP2K2*, the 12 candidate genes related to LS. A missense variant was found (c.1391G>G) in exon 12 (Gly464Ala) of the *PTPN11* gene, which led to the diagnosis of LS.

A beta-blocker was prescribed due to concerns about ventricular arrhythmias and LVOT obstruction associated with HCM-like pathophysiology. He had no arrhythmias noted on the ILR during the ∼1 year of observation, and there was no recurrence of syncope over the 2.5-year period.

## Discussion

We present a case of LS with coronary artery ectasia, AIVR, and SAM of the PMV with HCM-like pathophysiology. In previous epidemiological studies, the prevalence of congenital heart disease was 64%, and the majority of congenital heart diseases were HCM (71%), pulmonary artery stenosis (9%), and atrioventricular conduction defect (18%).^6^ There have been no reports of differences by sex, race, or ethnicity. Patients with LS who have left ventricular hypertrophy occasionally have stenosis of the LVOT. There is a case report regarding the complication of SAM of the PMV in NS, where it was hypothesized that the cause of SAM was the elongation of the middle scallop.^7^ Surgical treatment for these patients requires septal resection and valvuloplasty.

In the present case, the severity of the LVOT obstruction associated with SAM of the PMV was mild and did not require surgery. The patient was prescribed beta-blockers, and no arrhythmias were observed during the 1-year ILR monitoring period. Therefore, the syncope may have been caused by dehydration and aggravation of the LVOT obstruction due to working in humid and hot environment with a temperature of 104°F, humidity of 40% for 3 h. With regard to the PTPN11 variant, there was no family history of variant or sudden death, and a sporadic event was suspected. In this case, the patient was a solitary case and was diagnosed with LS for the first time in adulthood through a thorough examination following a syncope event. Some features of abnormal facial morphology, such as curly/coarse/sparse hair, hypertelorism, downward slanting palpebral fissures, ptosis, strabismus, broad flat nose, high arched palate, micrognathia, short webbed neck, and low-set posteriorly rotated ears, are less recognizable with age, and some PTPN11 gene variants are reported to be associated with less typical facial features, requiring caution during physical examination in adulthood.^2,3^ In addition, there were many morphological and electrophysiological abnormalities that may cause syncope, making it difficult to diagnose and determine treatment strategies. According to the ESC guidelines, the present case has a 5-year risk of SCD of 2.6%, and no ICD is recommended for primary prevention of syncope.^8^ In contrast, ILR is recommended in patients who do not have an indication for a primary prevention ICD or pacemaker if a comprehensive evaluation does not identify the cause of syncope, and no arrhythmia was noted after about 1 year of observation.^9^ Since sudden death has been reported in some cases, continuous careful observation is necessary.

## Conclusion

This report highlights cardiac complications underlying the pathogenesis of LS and management of patients. The present case is a report of a patient whose syncope led to the diagnosis of LS in adulthood for the first time, and is unique in that, in addition to left ventricular hypertrophy due to small myocyte collections and coronary artery ectasia, it has various cardiac complications such as AIVR and LVOT obstruction associated with SAM of PMV, which have not been reported previously. In current clinical practice, LS patients are often treated according to HCM guidelines as one of the secondary hypertrophic cardiomyopathies. However, the evidence for treatment is insufficient due to the small number of cases, and the extent to which drug or device therapy is effective has not been determined. More evidence should be accumulated to improve the management of these patients.

## Lead author biography


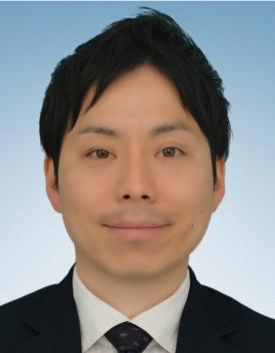


Naotoshi Wada, MD, PhD, graduated from Mie University in 2010 and completed doctoral course in medicine at Kyoto Prefectural University of Medicine in 2020. His primary areas of expertise include interventional cardiology, endovascular treatment, and heart failure.

## Data Availability

The data underlying this article are available in the article.
